# Large Language Model Prompting Techniques for Advancement in Clinical Medicine

**DOI:** 10.3390/jcm13175101

**Published:** 2024-08-28

**Authors:** Krish Shah, Andrew Y. Xu, Yatharth Sharma, Mohammed Daher, Christopher McDonald, Bassel G. Diebo, Alan H. Daniels

**Affiliations:** 1 Warren Alpert Medical School, Brown University, East Providence, RI 02914, USA; 2 Department of Orthopedics, Warren Alpert Medical School, Brown University, Providence, RI 02912, USA

**Keywords:** large language models (LLM), artificial intelligence (AI), prompt engineering, clinical decision support, medical innovation, healthcare technology

## Abstract

Large Language Models (LLMs have the potential to revolutionize clinical medicine by enhancing healthcare access, diagnosis, surgical planning, and education. However, their utilization requires careful, prompt engineering to mitigate challenges like hallucinations and biases. Proper utilization of LLMs involves understanding foundational concepts such as tokenization, embeddings, and attention mechanisms, alongside strategic prompting techniques to ensure accurate outputs. For innovative healthcare solutions, it is essential to maintain ongoing collaboration between AI technology and medical professionals. Ethical considerations, including data security and bias mitigation, are critical to their application. By leveraging LLMs as supplementary resources in research and education, we can enhance learning and support knowledge-based inquiries, ultimately advancing the quality and accessibility of medical care. Continued research and development are necessary to fully realize the potential of LLMs in transforming healthcare.

## 1. Introduction

Large language models (LLMs) are advanced natural language processing (NLP) models that analyze textual inputs to generate contextually relevant outputs. LLMs are trained on massive amounts of data from the internet using deep learning methods and can respond conversationally to an input from the user. Under the larger umbrella term of artificial intelligence (AI), LLMs are considered Generative AI, which is a category that describes data-generating AI, such as those which generate text, video, or audio [1]. Common examples of popular language models include GPT-3.5 and GPT-4, Jurassic, Dall-E, BERT, LLaMa, Gemini, and LaMDA [2].

LLMs are an enticing tool in the field of medicine due to the capability they possess to facilitate access to healthcare, diagnosis, and potential treatment options. Other applications include enhanced surgical planning, medical imaging accuracy, and physician–patient communication [3]. LLMs also provide researchers the ability to summarize and analyze vast amounts of articles in an expedited manner and filter large amounts of data [4]. However, if not provided an input that has a narrow range of response, LLMs can hallucinate, producing an output that is entirely fabricated and not grounded in existing knowledge [5]. Hallucinations are one of the many challenges that are encountered when utilizing LLMs in the healthcare field. Other challenges include LLMs having a perception and unfair bias towards certain populations, or misapplying valid knowledge to clinical situations [6]. Proper prompting techniques can mitigate these challenges by guiding LLMs to generate outputs closely aligned with relevant data and established knowledge, reducing the likelihood of hallucinations. By crafting prompts that explicitly account for diversity and specificity, biases can be minimized, and the risk of misapplying knowledge in clinical contexts can be significantly lowered [5]. Thus, strategic prompt engineering emerges as a crucial factor in enhancing the accuracy and fairness of LLM applications in healthcare.

A comprehensive overview will be provided regarding the correct utilization of LLMs with relevant examples through the detailing of proper prompting techniques. By doing so, we hope to educate clinicians about the proper use of this technology.

## 2. Background

Certain foundational technical concepts form the bedrock upon which LLMs’ remarkable linguistic and cognitive abilities are built. Knowledge of these concepts is required to better navigate the capabilities and limitations of the models [7]. This understanding is essential for harnessing the power of LLMs to support and advance research, clinical practice, patient education and beyond.

### 2.1. Tokenization

At the core of LLMs lies the process of tokenization, a mechanism that translates complex human language into a format that machines can understand and process. This procedure involves breaking down input text into smaller units, also called tokens, which represent the atomic elements of language understood by LLMs. For instance, a compound term like “notebook” might be segmented into “note” and “book,” demonstrating how the model parses compound words into constituent tokens. Each sentence, therefore, is understood by the model as a sequence of such tokens, ranging from individual words to punctuation marks. This granular breakdown into tokens allows LLMs to analyze and interpret the semantic nuances present within a text, facilitating a comprehensive understanding of the sentence as a whole [8].

In the operation of LLMs, a critical operational parameter is the token limit, which refers to the maximum number of tokens the model can process in a single input, or in simpler terms, the maximum text length for processing. This limit is inherent to the architecture and design of the model, influencing both its computational efficiency and its ability to understand and generate text. When a prompt exceeds this limit, it must be truncated or summarized before being fed into the model [9]. This constraint necessitates strategic consideration in the formulation of inputs, particularly in domains requiring the analysis of lengthy documents or comprehensive data, such as medical literature. The token limit impacts the model’s utility by requiring users to distill complex information into concise inputs, potentially affecting the depth of context or nuance the model can consider [10]. In practical applications, this means that while LLMs excel at processing and generating text within their token constraints, users must often employ summarization or selective focusing techniques to align with these limitations, ensuring that critical information is prioritized for analysis or task generation. Current token limits include 4096 tokens for GPT-3.5, while the paid version, GPT-4, has a token limit of 128,000 tokens [11]. As this technology advances, there will likely be increases in token limits and fewer constraints in text length for processing.

### 2.2. Semantic Understanding through Embeddings

LLMs transform tokens into high dimensional vectors through a process known as embedding. These vectors, or embeddings, encapsulate the semantic meanings of words as well as their positional information within sentences. A significant aspect of this embedding process is that vectors representing tokens with similar semantic information are positioned closely in the vector space, reflecting their conceptual similarity. This spatial arrangement facilitates the model’s ability to recognize and apply the nuanced relationships between words, enhancing its understanding of language patterns and context [12].

For example, within the domain of orthopedics, the terms “osteoporosis” and “osteopenia” refer to conditions characterized by bone density loss, albeit to different extents [13]. In the vector space, their embeddings would be positioned closely, reflecting their relatedness in the context of bone health disorders. This proximity enables the LLM to understand their semantic similarity and apply this knowledge in generating contextually relevant responses. Conversely, the term “osteoporosis” and a conceptually different term such as “myocardial infarction”, would have embeddings positioned further apart in the vector space [14]. Despite both being medical conditions, their embeddings reflect the significant difference in their semantic fields—bone health versus cardiac health. This distinction in the vector space allows the LLM to discern and process the vastly different contexts in which these terms are used, guiding the generation of accurate and context-specific outputs. This embedding mechanism underscores the LLM’s capacity to grasp and apply complex semantic relationships within specific domains such as medicine, enabling it to contribute effectively to tasks that require a deep understanding of specialized terminology and concepts [12].

### 2.3. Contextual Understanding through Attention

At the heart of modern LLMs, including the Generative Pre-trained Transformer (GPT) series, lies the transformer architecture. Transformers are a type of neural network that possess natural language processing, computer vision, and multi-model processing. These models are now the standard framework for constructing learning systems independent from recurrent neural networks and rigid architectures in language processing. This model architecture marks a departure from previous models that processed text sequentially. Instead, it analyzes sequences of tokens in parallel, significantly enhancing the model’s efficiency and its ability to grasp the nuances of context. The parallel processing capability allows for a more nuanced understanding of language, facilitating complex comprehension and generation tasks with unprecedented speed and accuracy [15].

A key facet of the transformer model is the encoder–decoder architecture, which consists of the encoder and decoder. The encoder is responsible for analyzing the input data, while the decoder delivers the output to the user, utilizing feedforward neural networks [15]. An important aspect of this relationship between input and output is the ability of the LLM to generate an output that is aligned with human expectations [16]. This is achieved by tuning. Tuning is the process by which a pre-trained LLM is adapted to consistently succeed on a specific task by training the model on a more concise labeled dataset specific to the target objective [17].

A cornerstone of the transformer architecture is its reliance on attention mechanisms, particularly self-attention. These mechanisms empower LLMs to evaluate the importance of different words within a sentence or across a document. By prioritizing certain tokens over others based on the prompt context, LLMs can generate responses that are both relevant and coherently aligned with the intended meaning. This ability to dynamically adjust focus within the text is what enables LLMs to produce highly accurate and contextually appropriate outputs, a feature of paramount importance in the precise and consequence-laden field of medicine [15].

A key model based on the transformer architecture is BERT (Bidirectional Encoder Representations from Transformers). BERT relies solely on the encoder portion of the transformer, meaning that it is designed to bidirectionally comprehend the words that come before and after a certain phrase. This ability is known as bidirectional contextual understanding and can be utilized to classify large-scale unlabeled text and answer questions [18].

### 2.4. Hallucinations

A “hallucination” is when faulty output is produced by the LLM that may seem evidence-based, but upon closer examination, it is often riddled with error-ridden information [19]. The main shortcoming to the application and utilization of LLMs in the greater healthcare field are “hallucinations” due to the adverse effects they can have such as misdiagnosis and incorrect information presented as facts [20]. Many studies suggest that LLMs frequently produce hallucinations, including factually incorrect references, which could significantly impact a researcher’s work if not properly acknowledged [21]. Thus, for LLMs to be utilized efficiently, it is imperative that users have a strong understanding of prompt engineering, specifically limiting ambiguities when providing input to LLMs.

## 3. Prompt Engineering

Utilizing prompting techniques is crucial for limiting hallucinations and ensuring time efficiency. Clear and concise prompts not only reduce ambiguity, enhancing the precision of information retrieval, but also result in quicker responses due to decreased processing time. These techniques significantly improve the user experience by minimizing delays, and are instrumental across all facets of medicine. Mastering effective prompting practices thus plays a key role in optimizing LLM outputs.

### 3.1. Prompt Design

At its core, prompt engineering seeks to optimize the structure and verbiage of the prompt, as the prompt is what contains the textual signals that steer the action of LLMs, influencing the ambiguities and factual inconsistencies within the output. Table 1 provides an overview of prompt design as well as relevant examples. A prompt consists of three main elements: instruction, output indicator, and context. The instruction component of the prompt presents a task that the LLM is to execute. This component usually begins with a verb. The output indicator provides the selected type or format of the output. The context provides extra information that helps shortlist the results of the LLM to more relevant outputs [22]. Not all prompts need to necessarily contain all three of these elements, depending on the task and the desired precision.

### 3.2. Zero-Shot, One-Shot, Few-Shot

Zero-shot, one-shot, and few-shot learning are terms that describe the ability of a model to understand and execute tasks with minimal examples or prior knowledge. Zero-shot learning refers to the model’s ability to correctly perform a task without any specific examples provided in advance. The model relies solely on its pre-existing knowledge and the instructions given in the prompt [23]. Table 2 provides an overview as well as relevant examples. One-shot learning involves providing the model with a single example to illustrate the task. This example serves as a pattern that the model can use to perform similar tasks [24]. Few-shot learning requires providing the model with several examples of the desired task or output. These examples help the model to better understand the nuances of the task and adjust its responses accordingly [24].

One- and few-shot learning approaches leverage the underlying capabilities of the LLM to adapt and generalize. The LLM deduces the preferred format and style by referencing the provided examples. The effectiveness of each approach can depend on the complexity of the task and the specificity of the domain. In general, more examples tend to give the model a better understanding of the task at hand, leading to more accurate and contextually appropriate outputs. However, even zero-shot learning can be surprisingly effective, particularly when the model has been well-trained in a specific domain or task type. For example, summarization will rarely require one-shot or few-shot learning.

### 3.3. Templating

Instead of providing examples or instructions, templating is a methodical approach to shaping the content and structure of model outputs [2]. By utilizing a predefined structure, templates ensure uniformity in the model’s responses. This method of templating can effectively supplant the need for one-shot or few-shot examples in situations where output variation is nominal, and the anticipated response can be aptly encapsulated within the confines of a template, allowing for consistent and precise responses.

Templates are constructed with varying degrees of sophistication, tailored to the complexity of the task at hand. Basic templating adopts a straightforward design, with placeholders—often denoted by brackets or other delimiters—where context variables are to be inserted. Complex templating provides a more elaborate framework for the LLM. It not only incorporates context variables, but is also imbued with additional logic layers, such as conditional statements that guide the generation process based on the input [2]. For instance, a template may contain branches that direct the content flow depending on whether certain conditions are met, thereby allowing for a dynamic response that aligns with the nuances of the input data. Additional abilities, such as a data integration point with Electronic Health Record (EHR) systems, are also possible, but require the use of the Open AI API, which is beyond the scope of this paper. Relevant clinical examples are provided in Table 3.

## 4. Applications for Medicine

### 4.1. Patient Communication

From direct conversations between patients and healthcare providers to indirect communications through Electronic Health Record (EHR) systems, patient reviews, and feedback mechanisms, communication is key to patient-centered care, yet it often imposes a significant time burden on healthcare professionals. Managing inquiries, responding to feedback, and ensuring effective communication can be time-consuming tasks that detract from direct patient care activities.

However, LLMs offer a transformative approach to patient engagement and communication in healthcare, given their potential to streamline or automate various forms of communications. This innovation significantly alleviates the administrative burden on hospital staff, enabling a more focused allocation of time and resources towards patient care and interactions that demand human expertise. Although direct patient management and diagnosis remain beyond the capabilities of LLMs, their strength lies in efficiently handling patient-initiated messages. LLMs excel in addressing routine patient inquiries related to treatment plans, recovery processes, and procedural logistics. By providing prompt and personalized responses that are aligned with individual patient profiles and medical histories, they enhance the quality of care and ensure information accessibility [25]. This strategic integration of LLMs into patient communication frameworks allows healthcare providers to focus on essential patient care while maintaining high communication standards, optimizing overall healthcare delivery. Currently, however, there are no FDA-approved devices that utilize generative AI for clinical communication with patients, as the focus remains on devices that integrate AI for tasks like image processing and triage notification management [26]. Thus, this gap underscores the evolving landscape of AI in healthcare, where significant potential exists for enhancing patient–provider interactions through integration of LLMs.

Additionally, LLMs can be configured to deliver tailored educational materials based on individual patient profiles, thereby enhancing understanding and engagement in the treatment process. This personalized approach can be complemented by automated follow-up and monitoring capabilities, which ensure continuous engagement with patients, prompt detection of potential complications, and timely adjustments to treatment plans [27]. Patawut et al. also demonstrated that LLMs can be used in analyzing patient feedback and reviews, underscoring the critical role of communication and perceived empathy in patient satisfaction [28]. Despite the varied impact of negative comments on traditional outcome measures, these studies highlight the importance of addressing patient concerns and improving the patient–clinician relationship. Furthermore, the use of NLP and sentiment analysis in examining patient comments and reviews, as seen in Khalid et al.’s work, validates the accuracy and utility of LLMs in gauging patient experiences and opinions, thereby offering a powerful tool for service enhancement based on direct patient feedback [29]. By systematically leveraging LLMs for feedback collection, healthcare providers can identify and address areas for improvement in patient–provider interactions and overall service delivery [30]. This comprehensive approach not only enhances patient satisfaction, but also contributes to better health outcomes, exemplifying the broader scope and potential of LLMs in revolutionizing patient engagement and communication within the healthcare sector.

Furthermore, LLMs possess the ability to alleviate a provider’s administrative obligations, which include documentation and writing clinical notes. LLMs can transform unstructured notes from a patient’s clinical record into a more structured format, simplifying documentation tasks in both routine patient care and clinical care trials which take up to 25% of a clinician’s time on average [7]. LLMs can also bridge the language barrier between a patient and a clinician through offering reliable, fast, and accurate translations, which allow for patients to comprehend the diagnostic process, regardless of the language they speak. Such multilingual capacity has recently been introduced in translating reports from other languages such as Spanish, Hindi, and Russian to English. More specifically, 50 deidentified abdomen-pelvis CT reports from the radiology archives at Mercy Fitzgerald Hospital were given to the ChaptGPT 4.0 model to translate, in which the translated English version showed no significant deviation from the Spanish and Russian versions in terms of factual correctness, potentially harmful errors, and completeness [31]. Thus, this approach of utilizing LLMs in multilingual settings demonstrates promise in providing greater transparency in patient–provider communication upon further training of the AI model.

### 4.2. Clinical Decision-Making

LLMs offer significant promise in supporting clinical decision-making, as they can sift through vast amounts of medical literature to provide evidence-based recommendations, summarizing the latest research findings and clinical guidelines, as well as assist in interpreting radiographic images and identifying relevant medical guidelines [32]. A recent LLM was developed for this specific purpose in the field of radiology, called Radiology-GPT. This LLM has been trained on a large, public dataset consisting of deidentified chest X-rays of nearly 60,000 patients from the Beth Israel Deaconess Center and is now capable of providing advice to clinicians on tasks such as preparing radiology reports and displaying clinical findings from an X-ray report [33]. This could support clinicians by offering a distilled view of current best practices and emerging treatments, streamlining the diagnostic process [34].

Furthermore, the application of LLMs extends to the operating room, as they have been shown to be effective in optimizing the selection of preoperative patients based on analysis of risk factors, planning out costs and resources needed for the hospital including length of stay and discharge for patients, and improving efficiency in surgical procedures regarding recommending techniques for the operative procedure [35,36]. However, while studies have shown LLMs like ChatGPT to be effective in diagnosing diseases based on symptom descriptions, their capabilities in proposing patient management strategies remain limited [25]. This stems primarily from the limited data set availability that LLMs can be trained on, which can lead to an output containing biased or highly inaccurate information [37]. The use of LLMs also raises ethical concerns, including patient privacy, data security, and model biases, necessitating transparent methodologies and strict oversight. Real-world integration of LLMs into healthcare also requires adherence to regulatory standards and validation to ensure AI-assisted decisions’ safety and reliability [25].

To fully harness the benefits of LLMs in healthcare, they should be integrated as part of a broader clinical decision-making framework. This approach ensures that LLMs enhance, rather than supplant, the expertise of medical professionals, providing a valuable tool in delivering comprehensive and informed patient care. To do so, clinicians must learn how to conduct proper prompt engineering in order to obtain the optimal support and guidance from LLMs. When utilizing prompt engineering correctly, clinicians have the ability to harness the power of LLMs to produce prompts specific to their needs [38]. Proper prompt engineering is the embedding of the task description that an AI intends to achieve within the prompt itself, often in the form of a question, instead of providing it explicitly. Such results can be achieved through providing a variety of prompts to the LLM, such as instructive prompts (in which the clinician can guide the LLM towards a specific task), system prompt (which provides context to the LLM for the input at hand), question–answer prompts (a question is asked to the LLM that is the emphasis of the input), and a mixed prompt (a combination of all the elements of the prompts aforementioned) [38].

### 4.3. Research and Education

LLMs have demonstrated mixed performance on medical specialty board exams. While more advanced models like GPT-4 have shown greater reliability, passing written sections of exams in fields such as general surgery, neurosurgery, orthopedics, and psychiatry, they have not passed exams in other specialties such as obstetrics and gynecology, gastroenterology, and radiology. Of note, earlier LLM models, such as GPT-3.5, have struggled more broadly with specialty board exams, but are still capable of passing the USMLE. These results underscore the variability in LLM capabilities, highlighting their strengths in knowledge recall and interpretation, but also their limitations in advanced judgment and complex reasoning. Consequently, LLM models should be used as supplementary resources in research and education to enhance learning and support inquiries focused on knowledge and interpretation.

LLMs are capable of revolutionizing education for medical students, residents, and clinicians by providing interactive learning tools. Whether supplementing resident training or continuing medical education (CME) programs, LLMs can create, and curate, education content tailored for each individual. LLMs enhance learning through creating simulation and case-based scenarios content, enabling learners to practice clinical reasoning [37]. Recently, AI Tutor, a web-based application, has become widely used through its utilization of (LLMs) to systematically curate an adaptive knowledge base [1]. This knowledge base is tailored to the specific nuances of the course, and constructed based on a comprehensive analysis of the input derived from the course materials [1]. The utilization of ChatGPT in surgical resident education during their trauma rotation has also been said to produce a more engaging and accessible learning environment, leading to increased standards of patient care and education [3].

Beyond their use in education, LLMs offer significant utility in research. Notably, they can greatly streamline literature reviews with their capability to efficiently sift through extensive medical literature and synthesize succinct summaries from each. An example of the literature search process being simplified through LLMs can be seen in the comparison between a PubMed search and a ChatGPT literature search. In a typical PubMed search, the user types in a query, and then the PubMed search engine seeks exact matches in the following indexed fields of the article: title, abstract, author list, keywords, and MeSH terms. In 2017, this search process was revised so that the most relevant articles were returned among the top results instead of the most recent articles. On the other hand, a ChatGPT literature search would be able to summarize the results of the literature search, provide answers directly to the user’s query through amalgamation of the results of the literature search, and provide recommendations for which literature to utilize to answer the user’s question. Furthermore, LLMs possess the capacity to explain the content of the literature to the user, thereby enhancing the user’s understanding of the content [39]. This ability enables students and researchers to distill key insights from a multitude of studies, facilitating a deeper understanding of prevalent themes and emerging trends.

With the advent of this transformative service comes concerns surrounding academic journals distancing themselves from plagiarism and false information that generative AI can sometimes produce. To address these concerns, students and researchers can take several measures, including clearly stating the way LLM tools were used in the research process, ensuring that human oversight was utilized in the final output, including fact-checking and utilizing as many open-source AI models as possible to ensure reproducibility of information [40]. To check hallucinations produced by the AI model, proper prompt engineering and cross-verification of AI-generated information with verified sources must be utilized to ensure that the information presented in the research piece is reliable [40]. Such actions will allow research to proceed in a positive manner through the added efficiency provided by LLMs.

In short, in order for students and clinicians to fully harness the benefits of LLMs in education and research, they should be incorporated within a comprehensive education system and research process [1].

## 5. Ethical Considerations

Patient data is highly sensitive, and any misuse or unauthorized access can lead to serious breaches of privacy. The protection of patient data requires robust measures to ensure that it is kept confidential and secure. Strong encryption methods are vital in safeguarding this information, making it difficult for unauthorized individuals to access or decipher the data. Adherence to regulations like the Health Insurance Portability and Accountability Act (HIPAA) is essential [8].

Ensuring informed consent is another critical aspect of protecting patient data. Patients must be fully aware of how their data will be used, stored, and shared. This transparency helps build trust and ensures that patients’ rights are respected. Employing robust anonymization techniques further safeguards patient information by removing or obfuscating personal identifiers, making it difficult to trace the data back to individual patients [41]. This is particularly important in research and data analysis, where the use of de-identified data can help protect patient privacy while still allowing valuable insights to be gained.

Closely related to patient privacy is data security, which involves preventing unauthorized access to or breaches of patient data. Comprehensive cybersecurity protocols are essential in this regard. These protocols include measures such as firewalls, intrusion detection systems, and regular security audits to identify and address potential vulnerabilities. Strict access control mechanisms are also crucial. By limiting access to sensitive health information to only those individuals who need it for their work, organizations can reduce the risk of unauthorized access [42].

Another significant ethical issue in the realm of patient data is the potential for large language models (LLMs) to perpetuate and even amplify existing biases present in their training data [43]. This can lead to unjust treatment of marginalized populations, which is misaligned with societal moral grounds and can further exacerbate health disparities [12]. For example, if the training data contains biases against specific racial or ethnic groups, the model might produce outputs that are prejudiced [44]. This can affect various aspects of healthcare, including diagnosis, treatment options, and patient outcomes. Such biases can result in some groups receiving substandard care or being misdiagnosed, which can have serious and long-lasting consequences, including misrepresentation of health outcomes and furthering health inequality between socioeconomic groups [42].

Furthermore, considerable declines in standards of safety and ethical consideration can occur in the realm of research when relying heavily on LLM assistance. Although such issues can be avoided through training of an LLM model, there can be uses of prompts designed to “jailbreak” the model, and thus avoid its inbuilt safety considerations [45].

To mitigate these risks, it is crucial to use diverse and representative datasets when training LLMs [46]. This helps ensure that the models are exposed to a wide range of scenarios and perspectives, reducing the likelihood of bias. Implementing rigorous bias detection and correction strategies is also essential. These strategies involve identifying potential biases in the models’ outputs and adjusting the models or their training data accordingly [47]. Continuous monitoring of LLM performance across different patient groups is another important step. By regularly assessing how the models perform for various populations, organizations can identify and address any disparities, ensuring that all patients receive fair and accurate treatment.

Protecting patient data is a multifaceted challenge that requires a combination of strong technical measures, adherence to regulations, and ethical considerations. Robust data protection strategies, such as encryption and anonymization, are essential in safeguarding sensitive information. At the same time, addressing the potential biases in LLMs is crucial to ensuring that all patients receive equitable care. By taking these steps, healthcare organizations can protect patient privacy, secure sensitive data, and provide fair treatment to all individuals, regardless of their background.

## 6. Conclusions

The integration of LLMs within healthcare represents a transformative shift towards leveraging AI to enhance healthcare delivery, clinical decision making, and medical education. Proper prompt engineering ensures that LLMs produce accurate, relevant, and contextually appropriate outputs, minimizing the risk of misinformation. The importance of continued growth is underscored here by detailing the unexplored area of strategic prompt engineering as a tool to mitigate the risks of hallucinations, biases, and the misapplication of knowledge in clinical contexts. Moreover, the adoption of zero-shot, one-shot, and few-shot learning, along with strategic templating, can further refine the utility of LLMs, making them more adaptable to the nuanced requirements of medical practice.

It is well known that LLMs can generate contextually relevant outputs based on vast training datasets, making them valuable assets in tasks such as automatic summarization and clinical decision-making. Yet, there are still many unknown factors, including how specific prompting techniques can systematically reduce risks such as hallucinations or biased information in clinical settings through practical applications. By clearly articulating these contributions, the current understanding of LLM applications is not only advanced, but actionable insights that can be directly applied in clinical practice are also offered, thereby paving the way for future research and AI-driven solutions in healthcare.

In conclusion, while challenges remain, the strategic application of LLMs in medicine presents a promising avenue for advancing healthcare. By fostering a collaborative relationship between AI technology and medical professionals, we can enhance the quality of care provided to patients and pave the way for innovative practices in both treatment and education. The future of medicine, enriched by AI, holds the promise of more efficient, accessible, and personalized care, underscoring the importance of continued research and development in this field.

## Figures and Tables

**Table 1:** Prompt Design Overview and Examples.

Element	Description	Example
Instruction	Presents a task that the LLM is to execute, usually begins with a verb.	Explain, Summarize, Translate, Generate, Compare, Create, Solve, Identify, Analyze, etc.
Output Indicator	Provides the selected type or format of the output.	Text, List, Table, Graph, Bullet Points, Code
Context	Enhances precision by adding extra details that guide LLM towards more relevant outputs.	Background information, Technical specifications, Length constraints, Tone, Style, Purpose, Audience

**Table 2:** Zero-shot, One-shot, Few-shot Overview and Examples.

Learning Type	Definition	Example
Zero-Shot	The model performs tasks without any specific examples, relying on pre-existing knowledge [23].	Generate a summary of an article without a prior example.
One-Shot	The model is given a single example to illustrate the task, using it as a pattern for similar tasks [24].	Diagnose a specific fracture type using an example radiograph.
Few-Shot	The model receives several examples to better understand the nuances of the task [24].	Identify various types of surgical tools with a few example images of each.

**Table 3:** Clinical Templating Examples.

Template Type	Example:
Basic	Post-Operative Instructions Template Wound Care: Keep the surgical site clean and dry. Cover the area with [Type of Dressing] and change the dressing every [Frequency]. Activity: Limit your activity to [Activity Level] for [Duration]. Avoid activities such as [Restricted Activities]. Medication: Take [Medication Name] for pain every [Dosage Interval] as needed. Begin [Antibiotic Name] the day after surgery and continue for [Duration]. Follow-Up: Schedule a follow-up appointment with your surgeon on [Follow-Up Date]. Additional Instructions: [Any Additional Instructions].
Advanced	Template for Patient Clinical Q&A: “Hello [Patient Name], thank you for reaching out with your concerns. You mentioned [Symptom/Condition]. Could you specify [Duration/Severity/Associated Symptoms]?” Conditional Logic for Symptom Assessment: If [Duration] > 2 weeks, then “Given the duration of your symptoms...” If [Severity] is high or [Associated Symptoms] include [List Severe Symptoms], then “These symptoms might suggest...” “Considering [Symptom/Condition] and [Duration/Severity/Associated Symptoms], the following steps are recommended...” Conditional Logic for Treatment Options: If [Symptom/Condition] is [Non-urgent Condition], then “Please consider the following treatment options…” If [Symptom/Condition] is potentially severe or requires immediate attention, then “It is important to seek immediate medical attention...” Follow-up Actions: “Please monitor your symptoms and [List Specific Actions].” “Should you experience [List of Warning Signs], seek immediate medical assistance.”

## Data Availability

Data sharing is not applicable to this article as no new data were created or analyzed in this study.
